# Experiences of nurses in managing HIV and Tuberculosis in rural clinics, South Africa

**DOI:** 10.4102/hsag.v30i0.3162

**Published:** 2025-11-13

**Authors:** Lwandile Tokwe, Portia J. Jordan, Regis R. Marie Modeste

**Affiliations:** 1 Department of Nursing and Midwifery, Faculty of Medicine and Health Sciences, Stellenbosch University, Cape Town, South Africa

**Keywords:** co-infection, HIV/pulmonary tuberculosis, primary health care, professional nurses, rural

## Abstract

**Background:**

Globally, an estimated 40.8 million people were living with Human Immunodeficiency Virus (HIV) in 2024, with approximately 8.15 million reported in South Africa by 2025. Pulmonary tuberculosis (PTB) remains a prevalent opportunistic infection among people living with HIV. There is a paucity of research on the experiences of professional nurses in managing HIV and PTB co-infection.

**Aim:**

This study aimed to explore and describe the experiences of professional nurses in managing newly diagnosed patients living with HIV and PTB co-infection in the rural primary health care (PHC) clinics.

**Setting:**

The research was conducted in Mthatha, Eastern Cape province, South Africa.

**Methods:**

A qualitative research approach with an exploratory, descriptive and contextual design was used. Data were gathered from 11 professional nurses through semi-structured interviews, which were audio recorded, transcribed, and analysed using Tesch’s eight-step method.

**Results:**

Three themes emerged, with Theme 1, exploring the roles of professional nurses in managing HIV and PTB co-infection. Theme 2, delved into the challenges experienced in managing this co-infection. Lastly, Theme 3 focused on the support structures that facilitated the care provision of patients.

**Conclusion:**

The HIV and PTB co-infection affects people globally. Tailored interventions are needed to support clinicians, especially professional nurses, in managing HIV and PTB co-infection and improving care for newly diagnosed patients.

**Contribution:**

This study provides insights into the experiences of professional nurses in managing newly diagnosed patients living with HIV and PTB co-infection. It further expands the knowledge on health care interventions adopted by professional nurses to promote the health of these patients.

## Introduction

Human Immunodeficiency Virus (HIV) is a global health concern, with an estimated 40.8 million people living with HIV (PLWH) and 1.3 million people newly diagnosed with HIV in 2024 (The United Nations Programme on HIV/AIDS 2025). In 2023, Eastern and Southern Africa reported 20.8 million People living with HIV, with South Africa carrying the largest burden of about 8.15 million People living with HIV (Statistics South Africa 2025; UNAIDS 2024). Despite the increased number of People living with HIV, pulmonary Tuberculosis (PTB) remains a common opportunistic disease that results in mortality in People living with HIV (Philipose et al. 2024). In addition, HIV is the common risk factor that predisposes latent PTB to develop into active PTB, and the risk is about 15–22 times higher than in people without HIV (World Health Organization [WHO] 2021). The WHO (2025) reported that PTB is the major cause of mortality associated with antimicrobial resistance in People living with HIV. In 2023, approximately 161 000 People living with HIV died from PTB worldwide (WHO 2025). Furthermore, the WHO (2024) reported that in 2022, PTB was the major cause of death in People living with HIV, accounting for 26% of the mortality rate in the African region. Moreover, Solomon et al. (2018) argued that mortality in HIV and PTB co-infection is highest in those with a CD4+ cell count less than 200 cells/µL. While this is noteworthy, Basu (2018) argued that HIV and PTB co-infection is part of South Africa’s broader quadruple burden of diseases.

Primary health care (PHC) facilities, the context of this study, are the first point of contact for individuals and families, providing health promotion, prevention, treatment and rehabilitation services (National Department of Health [NDoH] 2020a). In addition, the South African NDoH (2023a) emphasised that the PHC system serves as a vital connection between communities and health services and is predominantly nurse-led. Furthermore, Crowley et al. (2021) highlighted that professional nurses provide approximately 90% of health services in PHC clinics, suggesting the crucial role they play.

In 2013, South Africa’s PHC system implemented the ideal clinic initiative as an effort to enhance the quality of care provided (Muthelo et al. 2021). An ideal clinic refers to a clinic with adequate staff, suitable infrastructure, adequate medication and supplies, efficient administrative procedures and enough bulk supplies (Muthelo et al. 2021).

Currently, the HIV and PTB co-infection in the PHC clinics in South Africa is managed using fragmented clinical practice guidelines (CPGs) offered by the NDoH (2014, 2023a). However, scholars in this field highlight that the integrated management of HIV and PTB co-infection in PHC clinics is poor (Dlatu et al. 2023; Kalonji & Mahomed 2019). This leads to adverse patients’ outcomes, including loss to follow-up (LTFU), inadequate viral load suppression and, in severe cases, mortality. Furthermore, rural areas tend to have higher rates of certain diseases, because of socio-economic challenges and limited access to health care resources, which contribute to poorer health outcomes (Richman et al. 2019; Willie & Maqbool 2023). A study conducted in Limpopo province, South Africa, by Mutshatshi and Munyai (2022) highlighted critical challenges faced by PHC clinics, including staff shortages, inadequate equipment and supplies and infrastructure, as well as concerns regarding safety and security. Considering the essential role of professional nurses in delivering health care services, particularly in managing HIV and PTB co-infection in rural PHC clinics, exploring their experiences becomes crucial. The paucity of research on this topic underscores the necessity for further exploration. Therefore, this study aimed to explore and describe the experiences of professional nurses in managing newly diagnosed patients living with HIV and PTB co-infection in the rural PHC clinics.

## Research methods and design

A qualitative research approach with an exploratory, descriptive and contextual design was used. An exploratory design was appropriate because of the limited research on this topic. A descriptive design enabled detailed accounts of their experiences, while the contextual focus allowed the study to be grounded in a rural setting where professional nurses manage newly diagnosed HIV and PTB patients in rural PHC clinics.

### Setting

The study was conducted in four rural PHC clinics in Mthatha, located in Oliver Reginald Tambo District, within King Sabata Dalindyebo (KSD) sub-district in the Eastern Cape (EC) province of South Africa. In 2022, the setting had a population of approximately 476 558 (Census 2022). It comprises two towns, Mqanduli and Mthatha, and is predominantly inhabited by *isiXhosa*-speaking black people (Municipalities South Africa 2025). The sub-district has 44 clinics and five community health centres (KSD Municipality 2021).

### Sampling and sample

The purposive sampling method was used. The inclusion criteria were professional nurses managing newly diagnosed patients living with HIV and PTB in the rural PHC clinics. The exclusion criteria were professional nurses who were supporting the clinics and not necessarily permanent staff. The sample size included 11 professional nurses who consented to participate in the study. Three professional nurses were from PHC clinic one, while two were from PHC clinic two, two were from PHC clinic three and four were from PHC clinic four, which was a community health centre. The planned number was 15, and data saturation was reached at the 11th participant as there was no new information coming forth.

### Data collection method

Data were collected through semi-structured interviews conducted in *isiXhosa* by the researcher (first author), and an interview guide was used. The researcher recorded the interviews that were transcribed verbatim, then translated from *isiXhosa* into English by a language expert and verified by the first author. Field notes were also taken during the interviews.

### Data collection process

The PHC clinics were visited by the researcher to recruit participants and plan interviews. Meetings were held with PHC managers, during which printed copies of ethical approval letters and informed consent forms (ICFs) were provided. The PHC managers facilitated access to the professional nurses for information sharing and recruitment and arrangements were made with the interested participants.

The researcher used a combination of face-to-face and telephonic interviews with professional nurses. The researcher obtained participants’ consent to audio-record the semi-structured interviews, three of which were conducted telephonically. These three participants were available during the recruitment but not on duty on the day of the interviews. For the eight face-to-face interviews, the researcher scheduled early morning meetings at the clinics with the participants. The semi-structured interviews were conducted from the week of 15 May 2023 to 19 May 2023. The interviews were conducted in available consulting rooms or in the PHC managers’ offices. On the day of the interviews, the researcher requested the participants to sign an ICF. The semi-structured interviews with the professional nurses took about 45 min – 60 min each and were conducted in English and *isiXhosa*. The overall research question was: ‘Can you please share your experience of caring for newly diagnosed patients living with HIV and PTB co-infection?’

### Data analysis

The data analysis was conducted following Tesch’s eight data analysis steps (Creswell & Creswell 2018). The researcher coded *in vivo* and analysed the data. The independent coder used Atlas.ti version 2023 to code the data. After consultation and agreement between the first author and the independent coder, a consensus was reached. The authors then collaboratively began the analysis process until the themes emerged. A meeting was held to review all themes, during which some were renamed. Through this rigorous process, the themes and subthemes were finalised.

### Qualitative rigour

The qualitative rigour of the data was verified through Lincoln and Guba’s model of trustworthiness (1985), which comprises five criteria: credibility, dependability, confirmability, transferability and authenticity (Brink, Van der Walt & Van Rensburg 2018). Credibility was ensured by writing field notes while conducting interviews, member checking and peer debriefing. Dependability was promoted by peer examination. The methodology process was clearly explained to achieve an audit trail. Confirmability was guaranteed by prolonged engagement and member checking. Transferability was ensured by selecting the clinic participants based on the same criteria. Thick descriptions were also used; the researcher wrote clear inclusion criteria for the sampling method, recruitment process, data collection and the study’s research findings. Authenticity was ensured by writing field notes during each interview.

### Ethical considerations

This study was approved by the institutional Human Research Ethics Committee 1 (HREC1) (S22/09/175) in November 2022. Following that, the researchers sought approval in the EC province, and it was approved on 14 November 2024 (EC_202211_010). Lastly, the study was approved at the sub-district level in KSD on 17 November 2022. The face-to-face semi-structured interviews were conducted in private rooms to ensure privacy. Participants interviewed telephonically were requested to be in a private room to maintain confidentiality and ensure the integrity of the data collection process. Data were de-identified, and pseudonyms were used to maintain the anonymity of the participants. Confidentiality was ensured by not disclosing any shared information by participants. Participants were informed during the consent process that they would be compensated with a monetary token for their time and inconvenience. The researcher hereby acknowledges his position as an experienced PHC clinician who previously managed patients with HIV and PTB co-infection. During data collection, bracketing was practised. The researcher did not know the participants, and to reduce bias and coercion, participants were clearly informed that participation was voluntary and that they could withdraw at any time without affecting their care or dignity.

## Results

Table 1 presents the demographic details of the 11 participants in this study. A total of 10 female professional nurses and one male professional nurse participated in the study. Most participants had a bachelor’s degree in nursing (*n* = 6), while the rest had a 4-year comprehensive nursing diploma (Regulation 425). Two of the participants were specialists in primary care nursing. The participants had an average experience of 8 years, ranging from 2 to 25 years of experience in PHC. All the participants were Xhosa, and the interviews were conducted in *isiXhosa*.

**TABLE 1:** Professional nurses’ demography.

Pseudonym	Experience (years)	Qualifications
Pinky	8	Diploma in Community, Psychiatry and Midwifery Diploma in Primary Care Nursing
Sharika	15	Four-year Comprehensive Diploma in Nursing Diploma in Primary Care Nursing
Oney	5	Bachelor of Nursing (BCur) Master of Public Health Postgraduate Diploma in HIV Management
Thandeka	2	Diploma in General Nursing and Midwifery Diploma in Community Nursing
Thembelihle	6	BCur
Nokwakha	9	Diploma in Midwifery and General Nursing
Noma	25	Diploma in Midwifery Diploma in Community Health Nursing Diploma in Psychiatric Nursing Science BCur in Nursing Education and Management
Zee	8	Advanced Computer Literacy, Bachelor of Nursing
Lona	2	Four-year Comprehensive Diploma in Nursing
Anelisa	6	BCur
Flower	4	BCur

HIV, human immunodeficiency virus.

From the semi-structured interviews with the participants, three themes and nine subthemes emerged. The themes related to the roles in managing HIV and PTB co-infection and the challenges related to care provision and support structures are presented in Table 2.

**TABLE 2:** Themes and subthemes.

Themes	Subthemes
1. Roles in managing HIV and PTB co-infection	1.1 Managing the syndemic diagnosis in HIV and PTB co-infected patients 1.2 Adherence counselling, health education and routine monitoring 1.3 Realisation of the implications of co-infection with HIV and PTB
2. Challenges related to care provision	2.1 Resource constraints 2.2 Infrastructure and material constraints 2.3 Non-adherence to medication 2.4 Lack of training for nurses
3. Support structures	3.1 Community health workers (CHWs) assist with monitoring patients 3.2 Support from the stakeholders

HIV, human immunodeficiency virus; PTB, pulmonary tuberculosis.

### Theme 1: Roles in managing HIV/pulmonary tuberculosis co-infection

The first emergent theme focused on the roles of the participants in the management of newly diagnosed patients living with HIV and PTB co-infection. The roles involved screening, diagnosis of newly diagnosed patients living with HIV and PTB co-infection, conducting baseline investigations and HIV and PTB medication initiation. In addition, the participants expressed that they also conducted adherence counselling and provided health education. Furthermore, the participants also managed and facilitated routine monitoring of newly diagnosed patients living with HIV and PTB co-infection. Lastly, the participants verbalised that they realised the implications of the HIV and PTB co-infection on the newly diagnosed patients.

#### Subtheme 1.1: Managing the syndemic diagnosis in HIV/pulmonary tuberculosis co-infected patients

The participants described the management of newly diagnosed patients with HIV and PTB co-infection as an intricate process, beginning with comprehensive baseline investigations before initiating medication. This entailed collecting patients’ sputum specimens for PTB. The participants said:

‘Yes. At initiation for PTB, you collect the AFB sputum; then, after seven weeks, you do another one to change the patient to the continuation phase of the treatment; then, near the end of the treatment, you collect another one called the end of treatment smear.’ (Zee, female, 8 years’ experience)

‘For PTB, as I said, we start with GeneXpert as a baseline. Following that, we do the AFB, what we call a pre-treatment smear that is collected on the day of initiation.’ (Thembelihle, male, 6 years’ experience)

The participants expressed that there were other investigations they conducted, which focused on HIV diagnosis and monitoring. These included collecting blood specimens for CD4 count, syphilis testing and creatinine blood tests and sending them to the laboratory:

‘When we monitor HIV, a person will leave here having tested positive. We will then take the baseline blood as the CD4 count.’ (Pinky, female, 8 years’ experience)

‘Oh. Once the client is tested, we take blood, that is, the CD4 count, and their RPR, HB, what else, creatinine, and then yeah. Those are the baseline blood that we take when the patient has tested positive.’ (Anelisa, female, 6 years’ experience)

Interestingly, it became evident from the data that some of the participants omitted the standard baseline investigations. It was evident in the omission of collecting hepatitis B blood specimens and the cryptococcal antigen test by some of the participants. Although some of the omissions in the baseline investigations were noticed, participants expressed the importance of providing cotrimoxazole preventative therapy (CPT) on the day of the baseline investigations:

‘Yes. We also take hepatitis B. We take creatinine, hepatitis B, and Syphilis test for initiation of HIV.’ (Oney, female, 5 years’ experience)

‘Their CD4 counts are always low. We will then give them Cotrimoxazole; if they have PTB, they will be automatically put into it because they are taken to be coinfected.’ (Noma, female, 25 years’ experience)

‘And hence, our guidelines stipulate that if the CD4 count is less than 200, you administer prophylaxis because we understand that there are high chances of contracting PTB. Low CD4 count can result in PTB.’ (Thembelihle, male, 6 years’ experience)

Some of the participants highlighted the importance of monitoring patients’ vital signs, which include body weight, height and urinalysis, as part of the baseline data from newly diagnosed patients living with HIV and PTB co-infection:

‘I am checking weight, blood pressure [*BP*], height, BMI, it’s blood sugar and urine.’ (Thandeka, female, 2 years’ experience)

‘For HIV, since I’ve initiated on PTB, at three months, I take … On initiation, I take baseline blood. The baseline blood, which is the creatine, CD4 count, urine dipstick, if it’s a female, even a pregnancy test since you will notice that there is a contraindication in pregnant patients.’ (Oney, female, 5 years’ experience)

#### Subtheme 1.2: Adherence counselling, health education and routine monitoring

It is evident from the data that adherence counselling and education on initiation of medication were the core aspects implemented when managing the newly diagnosed patients living with HIV and PTB co-infection. The health education included adherence to medication and the correct time for taking medication. One of the participants stated that checking the readiness and comfort of newly diagnosed patients living with HIV and PTB co-infection before initiation to medication is of utmost importance:

‘We then try and teach them to take it right and also pay attention to the time they take it and not constantly change it.’ (Pinky, female, 8 years’ experience)

‘Then I educate the patient on how to take the medication for PTB; the treatment must be taken on an empty stomach when it comes to the stomach.’ (Thandeka, female, 2 years’ experience)

‘First of all, you ask about the comfortability of the patient in taking the medication and readiness to take the treatment.’ (Flower, female, 4 years’ experience)

The participants expressed the importance of providing health education, the possible side effects of the medication, as well as the possible strategies to improve lifestyle to newly diagnosed patients. One of the participants expressed that health education on proper diet is essential when initiating newly diagnosed patients living with HIV and PTB co-infection on medication:

‘As you start this pill, it’s the first time for you, and it will be new in your blood. You may have mild nausea. You may feel your body is not used to it, you may be tired.’ (Sharika, female, 15 years’ experience)

‘Yes, I educate for both HIV and PTB. Especially on PTB, it can be that red rash. It’s normal, then you know it’s going to end eventually and that change in colour of the urine. Others, they experience those things. Not that there are too many side effects.’ (Oney, female, 5 years’ experience)

‘We encourage them to grow in the garden so that someone will get vegetables because a healthy diet is to have vegetables in your food.’ (Sharika, female, 15 years’ experience)

The participants verbalised that the management of newly diagnosed patients living with HIV and PTB co-infection included managing the side effects that the patients experience from HIV and PTB co-infection medication:

‘And then with rash, we normally give, according to the guide, Chlorphenamine, that is, Allergex. When we give Rifafour, we give Pyridoxine for side effects.’ (Sharika, female, 15 years’ experience)

‘Otherwise, it is the colour of these pills. Nothing wrong. You have to drink a lot of water.’ (Sharika, female, 15 years’ experience)

‘If she takes medication after a meal, if the patient vomits, which is likely due to the number of the tablets, then when they vomit, one needs to educate the patient that they must check the vomitus and count the number vomited and retake the number that was vomited from his tablets again.’ (Oney, female, 5 years’ experience)

Some participants expressed that their management was based on the CPGs provided by the NDoH, such as antiretroviral therapy (ART) and adult primary care (APC) guidelines. What was concerning was the use of fragmented CPGs for managing side effects, which suggests a need for an integrated intervention for HIV and PTB co-infection. Although CPGs are provided to guide the management of patients in the clinics, this study found that the CPGs were outdated, which is a serious concern:

‘We do have guidelines, but of course, with skin rash, you just know that okay, this is what I can give to manage it. But there are guidelines.’ (Anelisa, female, 6 years’ experience)

‘There are guidelines from ART, from the national ART clinical guidelines that I am using and for PTB, there are also guidelines for reporting the adverse events, and there are guidelines about [*the*] management of those side effects or symptoms the patient is experiencing when taking the medication.’ (Flower, female, 4 years’ experience)

‘We do have a PTB guideline book, but it is old; it’s from 2014. I don’t usually see current ones. Ever since I started working, I don’t know what is happening.’ (Pinky, female, 8 years’ experience)

Interestingly, the participants shared different views regarding educating newly diagnosed patients living with HIV and PTB co-infection on routine monitoring. Some of the participants explained the process of the monitoring to the HIV and PTB co-infected patients. While some participants educated the newly diagnosed patients about the routine monitoring of HIV and PTB co-infection, others did not because of time constraints:

‘Yes. I educate the client about the blood, when the blood is going to be taken, and why they are taken in the first place.’ (Lona, female, 2 years’ experience)

‘I feel like we are not doing our main objective which is primary prevention. I feel like health education is not done in our clinics. That is what I really like; I’m not happy about that one. We don’t have time to do health education.’ (Anelisa, female, 6 years’ experience)

The participants found that managing newly diagnosed patients entailed monitoring them regularly once initiated on medication. The participants expressed that the patients were monitored using sputum and blood specimens collected at regular intervals, indicating the effectiveness or failure of the medication regimen for the management of the HIV and PTB co-infection. It is evident from the data that different HIV regimens have different intervals for monitoring blood. While the 3-month monitoring interval was associated with patients on a Dolutegravir (DTG)-based regimen, some of the participants monitored patients who were on a tenofovir, emtricitabine and efavirenz (TEE) regimen, which required them to collect blood at 6 months during medication:

‘I have to check this after three months, creatinine, then after six months, I have to repeat now, creatinine, CD4 and VL. When I see that the CD4 has increased, I stop Bactrim.’ (Thandeka, female, 2 years’ experience)

‘We take them on the first visit on initiation when they test positive, we take it, and then after three months we check the patient’s viral load whether he/she is virally suppressed, and then after six months and then yearly.’ (Anelisa, female, 6 years’ experience)

‘However, if it is HIV and PTB co-infection, at six months, we draw viral load and creatinine again. Then, at 12 months, we repeat viral load, CD4 count, and creatinine, then the patient is monitored yearly.’ (Noma, female, 25 years’ experience)

The participants not only expressed how they monitored for HIV but they also discussed how they monitored the HIV and PTB co-infection patients who were on PTB medication in their clinics:

‘Then after two months, at week seven, as it will be eight weeks, we take another AFB.’ (Oney, female, 5 years’ experience)

‘After seven weeks, the patient is called again for another sputum because we want to convert the patient to the continuation phase of the treatment.’ (Thembelihle, male, 6 years’ experience)

‘If the patient has been diagnosed with PTB by GeneXpert, we do the baseline, which is AFB. After seven weeks, we take another sputum for conversion.’ (Nokwakha, female, 9 years’ experience)

#### Subtheme 1.3: Realisation of the implication of HIV/pulmonary tuberculosis co-infection

The participants in this study stated that initiating newly diagnosed patients living with HIV and PTB co-infection on medication had negative implications, which they realised, including non-acceptance of the illnesses, stigma and fear of disclosure to the patient’s loved ones:

‘Mostly it is denial, our clients, they don’t want to accept at the initial phase of being diagnosed, that now HIV positive, they are PTB infected.’ (Lona, female, 2 years’ experience)

‘They do experience challenges, maybe a person will say they are not ready to disclose their status at home.’ (Pinky, female, 8 years’ experience)

‘And another thing is with this disclosure. They don’t want to disclose.’ (Sharika, female, 15 years’ experience)

While the issue of disclosure was mentioned by one of the participants, other participants found the issue of stigma experienced by newly diagnosed patients living with HIV and PTB co-infection:

‘Yes, they are still experiencing stigma because at first they fall sick and lose a [*lot*] of weight, by the look of things, when they are amongst other people or even when the client is still in the waiting area, other clients are just looking awkwardly at that client and the client feels ashamed at that time.’ (Lona, female, 2 years’ experience)

‘Eish, with HIV, stigma is still there. They are experiencing stigma. But with PTB, I don’t think it’s there.’ (Anelisa, female, 6 years’ experience)

‘Because they will think that their partner will stigmatise them or will judge them and say to them that maybe they are sleeping around when they are not present.’ (Flower, female, 4 years’ experience)

### Theme 2: Challenges related with care provision

The participants stated that caring for newly diagnosed patients living with HIV and PTB co-infection presented with challenges. These challenges were primarily based on resource constraints in the clinics essential to providing care to newly diagnosed patients living with HIV and PTB co-infection. In addition, some of the participants expressed the issue of unhealthy behaviours such as smoking and drinking alcohol, which hinder the efficacy of the medication prescribed for the patients.

#### Subtheme 2.1: Infrastructure and material constraints

The participants found that caring for newly diagnosed patients living with HIV and PTB co-infection is challenging because of the infrastructure resource constraints experienced in the PHC clinics, such as a lack of privacy because of limited consulting rooms, a poor ventilation system and a lack of waiting areas for the patients:

‘Especially if they are waiting for medicine, they will all stay here. Let alone that, we ask them to line up there, but when you look down, it’s outside. When the sun is hot, they get sunburned. When it rains, it rains.’ (Sharika, female, 15 years’ experience)

‘There is no privacy among patients here also. There is no nurse that is not sharing a consulting room. Sometimes you have four people in the room while you are talking to your client.’ (Nokwakha, female, 9 years’ experience)

‘Because as you can see, there is no ventilation system that filters that air quality and today it is cold, but we have to open the windows and the door.’ (Zee, female, 8 years’ experience)

A lack of equipment was another challenge reported in this study. Some of the participants expressed that caring for newly diagnosed patients living with HIV and PTB co-infection is challenging because of a lack of equipment, such as face masks, or stationery, such as clinic cards for carrying out administrative duties involving care. One of the participants expressed that they struggled to provide care because of issues such as information technology (IT) network connectivity, which is needed in the management of the patients, and stationery, such as paper, which affected the quality of care provision:

‘There are no masks, we use surgical masks, and there are no N95 masks. We were supposed to be using N95 masks. We don’t even have a sink to wash hands in the facility.’ (Zee, female, 8 years’ experience)

‘We only struggle with the network, which affects our X-ray machine.’ (Noma, female, 25 years’ experience)

‘You found out that there are no papers. And it is no longer dignified to write to the client on paper now.’ (Sharika, female, 15 years’ experience)

The participants stated that the clinics experience challenges with stockouts of the medication needed to manage PTB and the associated side effects, hindering their ability to provide care to the patients living with HIV and PTB co-infection:

‘Yes, sometimes a person may need a Rifinah, and we don’t have it, so we then ask another clinic. So that is a challenge.’ (Pinky, female, 8 years’ experience)

‘We experience stockouts, just like now we don’t have the vitamin B6 [*pyridoxine*], [*it*] is out of stock.’ (Zee, female, 8 years’ experience)

‘Yes, we do encounter such problems of drug stockouts, especially with HIV and PTB medication, but I think it is something that is more manageable because we do place our orders at monthly intervals.’ (Lona, female, 2 years’ experience)

However, while most participants expressed challenges related to medication shortages, a few found that they had medication in their clinics:

‘No, we always have stock in my facility. We don’t have instances whereby we say a patient did not get his or her TLD medication or Rifafour. No. They always get their medication.’ (Anelisa, female, 6 years’ experience)

‘We do have medication here for HIV and PTB, we have sputum jars.’ (Noma, female, 25 years’ experience)

#### Subtheme 2.2: Human resource constraints

The participants stated that the quality of the care provided to newly diagnosed patients living with HIV and PTB co-infection was poor because of the lack of human resources. The participants noticed that it is overwhelming to provide care if short-staffed, which affects the patient waiting times. While most participants highlighted the staff shortage challenge, one stated they were not short-staffed in their clinic:

‘Because of the flow here, I don’t think we provide quality care. I think we have quantity, not quality, because we don’t get that chance of sitting with the client for 30 minutes, you see.’ (Nokwakha, female, 9 years’ experience)

‘It gets overwhelming because there are a few nurses to attend to these people.’ (Pinky, female, 8 years’ experience)

‘In my facility, there are enough professional nurses.’ (Flower, female, 4 years’ experience)

Some of the participants expressed that their PHC clinics had a high volume of patients, which further impedes the health system regarding the quality of care provided to the participants:

‘So, with that waiting time, you find out that sometimes there is a rush to finish the patients.’ (Sharika, female, 15 years’ experience)

‘There is no waiting area for the clients. Even if it is raining, they stand there on the veranda. We don’t have a waiting area at all; even when it is cold or raining, they have to get all the cold outside. It is always full here.’ (Nokwakha, female, 9 years’ experience)

‘Even with our education, you have to rush and rush because you have other patients waiting for you on the veranda that are still looking at you.’ (Nokwakha, female, 9 years’ experience)

#### Subtheme 2.3: Non-adherence to medication

The participants verbalised that most patients living with HIV and PTB co-infection were non-adherent to their medication, as evidenced by the increased number of LTFU patients soon after they started feeling better from the medication for HIV and PTB co-infection:

‘Yes. They default. They easily default.’ (Oney, female, 5 years’ experience)

‘First, the patients we have, once they start the medication and see improvement in their health, they assume they are cured and stop taking their medication. Especially the patients who are first started on PTB mediation.’ (Thandeka, female, 2 years’ experience)

‘Yes, there are challenges with adherence. They do not; most of them do not come on their stipulated date for follow-up visits.’ (Flower, female, 4 years’ experience)

The participants further expressed challenges regarding the patients achieving healthy lifestyles, as some patients still engage in behaviours such as smoking and drinking alcohol, which affect their medication adherence. One of the participants further expressed that some patients come to the clinics intoxicated, which is quite concerning, as their health conditions become worse because of their unhealthy behaviours:

‘Some others default because of not caring, maybe by drinking and smoking, probably because of their living conditions.’ (Pinky, female, 8 years’ experience)

‘Yes, it is common in this community that I am living in that the patients are engaging in such behaviours while on medication such as drinking alcohol and smoking. Others even use snuff.’ (Flower, female, 4 years’ experience)

‘Umh … I feel okay. My clients, if they are not too sick, they don’t take it seriously; they still continue to drink their alcohol because they even come to the clinic drunk. They even come to the clinic smelling of a cigarette, and you can smell all of that inside the consulting room.’ (Anelisa, female, 6 years’ experience)

#### Subtheme 2.4: A lack of training for nurses

It became evident in the data that there was a lack of training for their day-to-day care provision. This is evidenced by how the government introduced CPGs and expected them to implement them in their respective PHC clinics without proper training, especially for HIV and PTB co-infection. The participants expressed frustration with not being supported in providing quality patient care:

‘My facility is the worst. Like there is no development there. Nurses are not learning anything. We are just there. We are just doing the work.’ (Anelisa, female, 6 years’ experience)

‘Yes, we have not received any training.’ (Lona, female, 2 years’ experience)

‘Yes, there is a shortage, there is a lack of training even in other non-communicable diseases, there is a lot of shortage because you would see when you are stuck, and you have to call someone or someone you know that they have this information.’ (Flower, female, 4 years’ experience)

One of the participants expressed concerns about her skills to manage PTB despite being competent in managing HIV, while others mentioned the scarcity of training in the PHC setting. One participant stated that she had to take the initiative to be trained and attend courses online despite being short-staffed. One of the participants expressed that while the training and support are limited in the PHC setting, they observed that some professional nurses find the CPGs time-consuming to use, even when available, and this limits the trust the patients have in them when they refer to the guidelines during a consultation:

‘I am talking more for HIV as compared to PTB. I have less management training when it comes to PTB. I am not that much properly trained on PTB. I am adequate on HIV management.’ (Thembelihle, male, 6 years’ experience)

‘Yho support … I support ha.a. In terms of training … Well, support is not that much. Like for instance, we have an APC guide as a clinician. It is the one that helps you [*with*] how to prescribe for this and how to diagnose this client. If you get a certificate, you apply online, study yourself and then get the certificate now. Otherwise, in-service training and many other things are rare nowadays.’ (Sharika, female, 15 years’ experience)

‘Yes, there are very few in-service trainings. So much so that some people are … Not all of us are easy to go to the guideline. Some people say that paging the guidelines is time-consuming. Some say that it lowers the confidence of you and the patient as you are consulting and looking at the book.’ (Oney, female, 5 years’ experience)

### Theme 3: Support structures

As a result of the heavy workload and staff shortage, the participants needed support at the PHC clinics. The participants expressed that they had external structures that supported them and the patients to ensure the provision of quality care to the newly diagnosed patients living with HIV and PTB co-infection. The support structures are in the form of teamwork in the clinics through assistance from the Community Health Workers (CHWs), the sub-district and support from the NDoH stakeholders such as Aquity Innovations and TB/HIV Care.

#### Subtheme 3.1: Community health workers assist with monitoring patients

The participants stated that some of the patient monitoring and screening in their clinics was supported by CHWs who work with the participants. This involved collecting sputum from patients, HIV screening and adherence counselling. The findings suggest teamwork between the clinicians and the ancillary staff regarding HIV and PTB screening and testing in the clinics:

‘So, HIV testing and PTB screening are mostly done by CHWs, we as professional nurses do it, but mostly but since there is a flock of clients, it is done by our lay counsellors (CHWs).’ (Lona, female, 2 years’ experience)

‘If the confirmation test is also positive, then the lay counsellor will send the patient back to me. I receive the patient from the lay counsellor as already positive. What I do then is to provide post-HIV counselling to this patient now that this patient’s HIV status is positive.’ (Thembelihle, male, 6 years’ experience)

‘A counselling was conducted by the lay counsellor when the client was being tested. So, in the consulting room, it is a continuation of that. We then counsel the client for PTB since the client will be taking a lot of tablets.’ (Nokwakha, female, 9 years’ experience)

#### Subtheme 3.2: Support from the stakeholders

The participants expressed that the support provided by the partners assists newly diagnosed patients living with HIV and PTB co-infection. The support partners include various organisations in the community, such as TB/HIV Care and Aquity Innovations. The participants expressed that the support came in the form of offering resources, adherence counselling or food to support newly diagnosed patients living with HIV and PTB co-infection. One of the participants expressed that the support for patients living with HIV and PTB co-infection is also ensured by contracting staff from local Non-Govenmental Organisations (NGOs) to trace HIV and PTB co-infection defaulters so that they can be retained in care:

‘We also get assistance in those cases by food parcels. We take the porridge from there and give it to them to eat.’ (Oney, female, 5 years’ experience)

‘Even us at Aquity, we have a PTB supporter who assists the patients with adherence counselling.’ (Zee, female, 8 years’ experience)

‘Yes, the ones who drink alcohol, and you know we have a colleague from the NGO who does tracing, and she finds most of the patients in the tavern.’ (Thandeka, female, 2 years’ experience)

Some of the participants highlighted the assistance of CHWs and adherence counsellors from the NGOs in ensuring that adherence counselling on medication initiation is provided to patients living with HIV and PTB co-infection:

‘We meet up with our NGO; there is a TB/HIV Care NGO where we meet up with health villagers like CHWs to discuss the particular person and give them their contact details because they will be able to check on the client by first calling them.’ (Pinky, female, 8 years’ experience)

‘Yes, and then from there, we also have counsellors from the Department of Health and the NGOs we are working with that assist us with the adherence counselling.’ (Flower, female, 4 years’ experience)

‘But we also work with TB/HIV Care, which is another NGO that has adherence counsellors. So, the patients who test positive for HIV undergo adherence counselling by the adherence counsellor at TB/HIV Care.’ (Zee, female, 8 years’ experience)

The participants verbalised that the support partners assisted them with medical resources such as suctioning machines, staff and even medication to enhance the care provided to newly diagnosed patients living with HIV and PTB co-infection:

‘We have Aquity Innovations staff. They are here for sputum collection for our patients. They assist them. Let’s go to them. And they also have a suctioning machine like these who can’t produce us, they suction them. Then we say that he should come back.’ (Oney, female, 5 years’ experience)

‘Yes, we do have NGOs such as TB/HIV Care. They are always there, especially when it comes to HIV and PTB medication, clients for the co-infection getting lost, that is, your lost to follow-ups, and early and late defaulters. We do get a lot of support from those NGOs. Yes.’ (Lona, female, 2 years’ experience)

‘They also have their own CHWs that assist when we have lost to follow-ups. They assist us with that. TB/HIV Care also has a data capturer, people who will make sure that these people are captured [*and*] appear on Tier.net.’ (Oney, female, 5 years’ experience)

## Discussion

To the researchers’ knowledge, this is the first study to explore the experiences of professional nurses managing newly diagnosed patients living with HIV and PTB co-infection, especially in the rural PHC clinics. The participants in this study highlighted their role in HIV and PTB screening and testing, medication initiation for HIV and PTB co-infection, monitoring and managing side effects. Similar to these findings, the South African NDoH (2020b) highlighted that professional nurses’ roles include screening for PTB and HIV testing, initiating patients on medication, providing adherence counselling, monitoring and managing side effects. A study conducted by Phetlhu et al. (2018) highlighted that one of the roles of professional nurses in PHC clinics is to ensure that the HIV status of a new patient is known prior to sending the sputum to the laboratory for PTB diagnosis.

The participants in this study provided health education and adherence counselling to the newly diagnosed patients on the day of initiation of medication for HIV and PTB. A study by Ticha, Bimerew and Phetlhu (2022) highlighted that PHC nurses have roles in managing HIV and PTB co-infection, which includes health education and adherence counselling. The findings presented are similar to the findings of this study regarding the roles of professional nurses in managing HIV and PTB co-infection.

This study found that the PHC clinics examined had resource constraints in terms of infrastructure, medication for HIV and PTB co-infection and shortage of staff needed for smooth operation. The challenge of resource constraints, particularly HIV and PTB medication in the PHC clinics, is a long-standing challenge and has been reported in studies conducted in 2011 and 2014. Loveday and Zweigenthal (2011) highlighted that the PHC clinics in their study experienced medication stockouts for ART and PTB treatment as indicated by 80% and 30% of clinics, respectively, which potentially reduced the efforts of integration of HIV-TB management. Similarly, a report by Stop Stock Outs in South Africa (2014) highlighted that rural clinicians in the PHC clinics and other levels of care face enormous frustration because of essential medicine stockouts. The stockouts of medication, especially PTB medication, which includes pyridoxine and others, were evident in the findings of this study.

This study found that staff shortages contribute to participants providing poor-quality service. The shortage of health personnel in the health system is a global problem (WHO 2016). The WHO (2022) reported that the shortage of health workers in Africa will be about 6.1 million by 2030, a 45% increase from 2013 projections. Mutshatshi and Munyai (2022) and Stime et al. (2018) suggested that a shortage of staff in the PHC clinics is stressful to the staff and is associated with poor staff morale, long waiting times for the patients and poor patient satisfaction, which may contribute to non-adherence to medication and poor clinic attendance. According to Adeniji and Mash (2016), cited in Abrahams (2021), PHC clinics are usually poorly resourced, and challenges such as staff shortages are common. In addition, when a shortage of staff is prevalent in the clinics, patients experience long waiting times, leading to low patient satisfaction (Abrahams, Thani & Kahn 2022).

The challenge of non-adherence of newly diagnosed patients living with HIV and PTB co-infection was also highlighted by the participants. The findings reveal that some patients default on their medication as soon as they begin to feel better. Supporting this result, Cameia et al. (2020) reported that some patients default on their medication when they physically improve and their symptoms disappear because they think they are cured.

The participants reported a lack of training, particularly in the skills needed to manage newly diagnosed patients living with HIV and PTB co-infection. It was further found that the CPGs would be delivered without proper training in some cases, affecting the implementation of HIV and PTB programmes. According to Melariri, Kalinda and Chimbari (2021), training the health care team empowers them with the knowledge and attitude necessary for effective practice. A study by Mboweni and Makhado (2020) highlighted that professional nurses working in the PHC clinics experience frustration with their inability to initiate Highly Active Antiretroviral therapy (HAART) on patients because of a lack of training. In addition, a study by Ticha et al. (2022) found that there is a great need to equip professional nurses caring for patients living with HIV and PTB co-infection with adherence counselling skills to improve practice. A study by Phetlhu et al. (2018) revealed that some clinicians, particularly those in rural areas, receive limited or no training on HIV and PTB integrated care and lack the knowledge needed to manage HIV and PTB co-infection. Similarly, Anyebe et al. (2021) highlighted that a lack of training is a significant challenge in providing care in a PHC setting. Therefore, the findings of this study are consistent with those reported in the literature.

Participants in this study revealed that support from partners such as TB/HIV Care and Aquity Innovations assisted the management of newly diagnosed patients with HIV and PTB co-infection. A published South African commentary study conducted by Pillay (2022) revealed that NGOs continue to support the NDoH in various areas by employing different categories of health workers, providing stipends to community health workers and offering training and capacity-building, including in the implementation of new clinical guidelines and training of CHWs. However, Ndjeka et al. (2025) pointed out that recent funding cuts from the United States have had a substantial impact on PTB services in South Africa. In addition, Venter (2025) found that these US-funded NGO clinics led to shortages of ART and job losses among various health care workers involved in HIV programmes in South Africa. As a result, it is recommended that the South African NDoH should prioritise sustaining the essential services that were provided by NGOs within PHC clinics.

### Strengths and limitations

The strength of this study lies in the exploration of the experiences of professional nurses managing patients living with HIV and PTB co-infection in the rural PHC clinics of South Africa. The qualitative data were collected by the researcher and analysed by all authors. To reduce bias, an independent coder was also engaged. A key limitation of the study is that semi-structured interviews were conducted in only one sub-district within the OR Tambo District.

## Conclusion

The primary objective of this study was to explore and describe the experiences of professional nurses in managing newly diagnosed patients living with HIV and PTB co-infection in the rural PHC clinics. The objective of this study was achieved. The findings of this study show that professional nurses take on multiple roles that manage newly diagnosed patients living with HIV and PTB co-infection. Despite the crucial role played by professional nurses, this study’s findings affirm that there is poor integration of health services in the rural PHC clinics. The findings of this study further demonstrate that professional nurses experienced human, infrastructure and material resource constraints in the PHC clinics, which affected the quality of nursing care provided. Lastly, the findings revealed that the assistance of support structures such as NGOs and CHWs assisted professional nurses in making the journey of newly diagnosed patients easier in the PHC clinics. It is hoped that these findings may inform the development of interventions to support the health care team in the PHC clinics on the management of HIV and PTB co-infection in the rural PHC clinics.
